# Supplementary material for: The Relative Preservation of the Central Retinal Layers in Leber Hereditary Optic Neuropathy
Source: J Clin Med. 2022 Oct 13;11(20):6045. doi: 10.3390/jcm11206045 (PMC9604528; doi:10.3390/jcm11206045)
Supplement: Supplementary file 1 [file jcm-11-06045-s001.zip › Supporting Table S1 JCM.pdf]

Table S1. List of 35 genes included in clinical exome next generation sequencing panel as possible mutational spots for hereditary optic nerve diseases

	Gene
1	ACO2
2	MFN2
3	NR2F1
4	OPA1
5	OPA3
6	RTN4IP1
7	SLC25A46
8	SLC52A2
9	WFS1
10	C12orf65
11	C19orf12
12	CISD2
13	DNM1L
14	MFF
15	SPG7
16	AFG3L2
17	POLG
18	TIMM8A
19	TMEM126A
20	C12ORF65
21	NDUFS1
22	SSBP1
23	ATAD3A
24	DNAJC19
25	OPA6
26	FXN
27	AUH
28	MTPAP
29	YME1L1
30	TSFM
31	OPA8
32	ZNHIT3
33	OPA4
34	OPA2
35	DNAJC 30
