# Supplementary material for: The Relative Preservation of the Central Retinal Layers in Leber Hereditary Optic Neuropathy
Source: J Clin Med. 2022 Oct 13;11(20):6045. doi: 10.3390/jcm11206045 (PMC9604528; doi:10.3390/jcm11206045)
Supplement: Supplementary file 1 [file jcm-11-06045-s001.zip › Supporting Table S2 JCM.pdf]

Table S2. Patient basic phenotype and genotype data

Patient ID	Date_of_birth	Sex	Genetic analysis	Eye	Date of onset	Age at the study	Age at onset	Time onset-study	Color vision (Ishihara)	Visual acuity (Snellen)	PERG N95	VEP P100	Visual field (VF) defect	VA improvement
LHON 1	26/2/59	M	MT-ND4:m.11778G>A, homoplasmy	RE	1/3/16	60.38	57.01	3.37	0/15	0.01	Reduced, in the level of the baseline	Reduced and delayed	Small sensitivity island in temporal VF preserved	No
				LE	1/3/16	60.38	57.01	3.37	0/15	0.01	Reduced, in the level of the baseline	Undetectable	Small part of sensitivity in temporal VF preserved	No
LHON 2	8/12/85	M	MT-ND1: m.3460G>A homoplasmy	RE	15/10/11	33.92	25.85	8.07	0/15	0.015	Reduced, in the level of the baseline	Reduced and delayed	Central scotoma	No
				LE	1/11/11	33.92	25.90	8.03	0/15	0.01375	Reduced, in the level of the baseline	Reduced and delayed	Central scotoma	No
LHON 3	13/11/56	M	MT-ND4:m.11778G>A, homoplasmy	RE	Childhood Amblyopic				0/15	0.001	Reduced, above the baseline	Undetectable	Central scotoma	No
				LE	15/1/17	61.13	60.17	0.96	0/15	0.001	Reduced, above the baseline	Undetectable	Central scotoma	No
LHON 4	25/10/89	M	MT-ND6: m14484 T>C, p.Met64Val homoplasmy	RE	1/6/10	28.35	20.60	7.75	6/15	1	Reduced, above the baseline	Reduced, borderline delayed	Small central scotoma	Yes
				LE	Childhood Amblyopic				1/15	0.1	Reduced, above the baseline	Reduced, borderline delayed	Without large scotomas	No
LHON 5	20/7/01	M	MT-ND4:m.11778G>A, 92 % heteroplasmy	RE	13/12/17	16.61	16.40	0.21	0/15	0.02	Reduced	Reduced and delayed	Central scotoma	No
				LE	13/12/17	16.61	16.40	0.21	0/15	0.05	Reduced	Reduced and delayed	Central scotoma	No
LHON 6	16/12/64	F	MT-ND1: m.3460G>A homoplasmy	RE	1/1/87	55.62	22.04	33.58	0/15	0.0125	NA	NA	Central scotoma	No
				LE	1/1/87	55.62	22.04	33.58	0/15	0.0125	NA	NA	Central scotoma	No
LHON 7	19/8/75	F	MT-ND1: m.3460G>A homoplasmy	RE	1/1/94	45.03	18.37	26.66	0/15	0.005	NA	NA	Central scotoma	No
				LE	1/1/94	45.03	18.37	26.66	0/15	0.0125	NA	NA	Central scotoma	No
LHON 8	16/10/57	F	MT-ND1: m.3460G>A homoplasmy	RE	1/1/67	62.92	9.21	53.71	0/15	0.005	Reduced, in the level of the baseline	Undetectable	Isopter II/4 narrowed 10-15°, II/3 narrowed	No
				LE	1/1/67	62.92	9.21	53.71	0/15	0.005	Reduced, in the level of the baseline	Undetectable	Isopter II/4 narrowed 20-30°, II/3 10-15°, concentric VF narrowing	No
LHON 9	15/7/77	F	MT-ND1: m.3460G>A homoplasmy	RE	1/1/88	43.17	10.46	32.71	15/15	0.6	Reduced, in the level of the baseline	Reduced, borderline delayed	Central scotoma	Yes
				LE	1/1/88	43.17	10.46	32.71	15/15	0.6	Reduced, in the level of the baseline	Reduced, borderline delayed	Central scotoma	Yes
LHON 10	15/10/98	M	MT-ND1:m.3700G>A, homoplasmy	RE	Childhood Ambliopic				0/15	0.015	Reduced, above the baseline	Undetectable	Centrocecal scotoma	No
				LE	27/6/05	20.69	6.70	13.99	1/15	0.05	Reduced, above the baseline	Undetectable	Centrocecal scotoma	No
LHON 11	13/8/84	M	MT-ND5, m.13042G>T homoplasmy	RE	1/9/04	28.39	20.05	8.33	0/15	0.005	Reduced, above the baseline	Undetectable	Central scotoma	No
				LE	27/7/04	28.39	19.95	8.43	0/15	0.005	Reduced, above the baseline	Undetectable	Central scotoma	No

LHON 12	4/9/95	M	MT-ND5, m.13042G>T homoplasmy	RE	22/11/06	20.66	11.22	9.44	1/15	0.015	Reduced, above the baseline	Undetectable	Central scotoma 10-20°	No
				LE	22/11/06	20.66	11.22	9.44	1/15	0.015	Reduced, above the baseline	Undetectable	Central scotoma 10-20°	No
LHON 13	11/7/91	M	DNAJC 30 152 A>G (p.Tyr51Cys)	RE	1/8/09	25.48	18.06	7.42	15/15	0.7	Relatively reduced, above the baseline	Normal amplitude, borderline delayed	Central scotoma with fenestrations LV 37.7	Yes
				LE	1/8/09	25.48	18.06	7.42	15/15	0.7	Relatively reduced, above the baseline	Normal amplitude, borderline delayed	Central scotoma with fenestrations LV 52	Yes
LHON 14	9/10/89	M	DNAJC 30 152 A>G (p.Tyr51Cys)	RE	1/8/07	22.01	17.81	4.21	2/15	0.03	Reduced, in the level of the baseline	Undetectable	Central scotoma, within 10 deg	No
				LE	14/8/07	22.01	17.85	4.17	2/15	0.03	Reduced, in the level of the baseline	Undetectable	Central scotoma within 20 deg	No
nonLHON 1	30/5/60	F	No pathogenic variants in mtDNA or WES	RE	1/9/18	58.67	58.26	0.42	1/15	0.3	Slightly reduced	Normal amplitude, abnormal shape	Central scotoma, concentric VF narrowing	No
				LE	1/9/18	58.67	58.26	0.42	1/15	0.2	Slightly reduced	Normal amplitude, delayed	Central scotoma and concentric VF narrowing	No
nonLHON 2	16/2/61	M	No pathogenic variants in mtDNA or WES	RE	1/1/10	57.75	48.87	8.88	0/15	0.0125	Reduced	Undetectable	Relative central scotoma, II1 and II2 ispoters undetectable, blind spot on II4	No
				LE	1/1/10	57.75	48.87	8.88	0/15	0.0125	Reduced	Undetectable	Relative central scotoma, II1 and II2 ispoters undetectable, blind spot on II4	No
nonLHON 3	1/1/63	M	No pathogenic variants in mtDNA or WES	RE	1/1/77	49.92	14.00	35.91	0/15	0.2	Reduced, in the level of the baseline	Reduced, normal latency	Centrocoecal scotoma	No
				LE	1/1/10	57.75	48.87	8.88	0/15	0.03	Reduced, above the baseline	Significantly reduced, slightly delayed, W shape	Centrocoecal scotoma	No
nonLHON 4	12/11/90	M	No pathogenic variants in mtDNA or WES	RE	1/1/77	49.92	14.00	35.91	1/15	0.0125	Reduced, above the baseline	Reduced and delayed	Centrocoecal scotoma between 10 and 20 deg	No
				LE	1/1/77	49.92	14.00	35.91	1/15	0.15	Reduced, above the baseline	Reduced and delayed	Centrocoecal scotoma	No
nonLHON 5	11/7/80	F	No pathogenic variants in mtDNA or WES	RE	1/4/13	36.22	32.72	3.50	0/15	0.03	Reduced, above the baseline	Significantly reduced, delayed, W shape	Central scotoma	
				LE	1/4/13	36.22	32.72	3.50	0/15	0.04	Reduced, above the baseline	Significantly reduced, delayed, W shape	Central scotoma	No
nonLHON 6	15/3/71	F	No pathogenic variants in mtDNA or WES	RE	1/4/99	41.89	28.05	13.84	0/15	0.2	Reduced, above the baseline	Reduced and delayed	Central and inferior visual field scotomas	No
				LE	1/4/99	41.89	28.05	13.84	0/15	0.2	Reduced, above the baseline	Reduced and delayed	Central scotoma	No
nonLHON 7	28/3/86	F	No pathogenic variants in mtDNA or WES	RE	1/1/12	32.51	25.76	6.75	0/15	0.5	NA	NA	Decreased sensitivity centrally	No
				LE	1/1/12	32.51	25.76	6.75	0/15	0.5	NA	NA	Decreased sensitivity centrally	No
nonLHON 8	30/3/60	F	No pathogenic variants in mtDNA or WES	RE	1/1/70	58.50	9.76	48.75	0/15	0.1	Reduced, above the baseline	Normal amplitude and latency, abnormal shape	Concentric VF narrowing, central decrease of sensitivity	No
				LE	1/1/70	58.50	9.76	48.75	0/15	0.1	Reduced, above the baseline	Normal amplitude and latency, abnormal shape	Concentric VF narrowing, central decrease of sensitivity	No

nonLHON 9	4/1/99	M	No pathogenic variants in mtDNA or WES	RE	5/3/01	19.27	2.17	17.11	0/15	0.015	Reduced, in the level of the baseline	Reduced and delayed	Central scotoma	No
				LE	5/3/01	19.27	2.17	17.11	15/15	1	Slightly reduced	Normal amplitude and latency, abnormal shape	Slightly decreased sensitivity centrally	Yes
nonLHON 10	17/4/64	M	No pathogenic variants in mtDNA or WES	RE	1/12/06	52.79	42.62	10.17	0/15	0.001	Reduced	Undetectable	Central scotoma	No
				LE	1/12/06	52.79	42.62	10.17	12/15	1	Reduced, in the level of the baseline	Normal amplitude and latency, abnormal shape	Nasal and central scotoma	Yes
nonLHON 11	28/4/89	F	No pathogenic variants in mtDNA or WES	RE	1/1/00	25.34	10.68	14.67	1/15	0.16	Reduced, in the level of the baseline	Reduced and delayed	Reduced sensitivity in whole visual field	No
				LE	1/1/00	25.34	10.68	14.67	1/15	0.5	Reduced, in the level of the baseline	Borderline reduced and delayed	Reduced sensitivity in whole visual field	NO
nonLHON 12	19/9/36	M	No pathogenic variants in mtDNA or WES	RE	1/4/16	83.70	79.53	4.17	1/15	0.3	NA	NA	Concentric VF narrowing, central decrease of sensitivity	No
				LE	1/4/16	83.70	79.53	4.17	0/15	0.4	NA	NA	Concentric VF narrowing, central decrease of sensitivity	No
nonLHON 13	9/3/59	M	No pathogenic variants in mtDNA or WES	RE	1/8/14	55.90	55.40	0.50	1/15	0.2	Reduced, in the level of the baseline	Reduced and delayed	Central scotoma, reduced VF sensitivity	No
				LE	1/8/14	55.90	55.40	0.50	5/15	0.4	Reduced, in the level of the baseline	Reduced and delayed	Central scotoma, reduced VF sensitivity	No
