# Supplementary material for: The Relative Preservation of the Central Retinal Layers in Leber Hereditary Optic Neuropathy
Source: J Clin Med. 2022 Oct 13;11(20):6045. doi: 10.3390/jcm11206045 (PMC9604528; doi:10.3390/jcm11206045)
Supplement: Supplementary file 1 [file jcm-11-06045-s001.zip › Supporting Table S3 JCM.pdf]

Table S3. Demographic characteristics of patients included in this study.

Variable	LHON	nonLHON	p
male: f (%)	10 (71.14 %)	7 (54 %)	0.873
left eye			
time from onset (years): mean (SD)	14.2 (16.0)	12.4 (15.0)	0.782
age (years): mean (SD)	37.8 (18.1)	48.2 (16.7)	0.157
n	12	12	/
right eye			
time from onset (years): mean (SD)	16.3 (16.5)	13.5 (15.5)	0.673
age (years): mean (SD)	36.9 (16.1)	48.7 (17.9)	0.113
n	12	11	/
