# Supplementary material for: The Relative Preservation of the Central Retinal Layers in Leber Hereditary Optic Neuropathy
Source: J Clin Med. 2022 Oct 13;11(20):6045. doi: 10.3390/jcm11206045 (PMC9604528; doi:10.3390/jcm11206045)
Supplement: Supplementary file 1 [file jcm-11-06045-s001.zip › Supporting Table S4 JCM.pdf]

Table S4. Electrophysiology data showing no significant difference ($p > 0.05$) in PERG and VEP. it-implicit time, amp-amplitude

	LHON (average ± SD)	NonLHON (average ± SD)	*p*
it P50 ms	47.9 ± 3.35	46.00 ± 3.45	0.196
amp P50µV	4.1 ± 1.10	3.89 ± 1.64	0.804
it N95 ms	101.7 ± 8.65	105.08 ± 10.45	0.563
amp N95 µV	3.77 ± 0.83	3.89 ± 1.58	0.662
it P100 ms	137.5 ± 18.48	127.11 ± 29.05	0.529
amp P100 µV	1.51 ± 2.47	4.49 ± 5.13	0.073
it P100 ms	129 ± 24.43	105.66 ± 45.19	0.423
amp P100_RHF µV	1.6 ± 1.25	2.63 ± 2.43	0.333
it P100_LHF ms	131.5 ± 21.92	109.67 ± 47.53	0.554
amp P100_LHF µV	2.55 ± 0.49	3.56 ± 3.29	0.688
it FERG_wave_ a ms	17.2 ± 2.94	16.25 ± 3.19	0.383
amp FERG_wave_a µV	37.29 ± 18.92	33.98 ± 16.17	0.562
it FERG_wave_b ms	41.8 ± 3.26	41.00 ± 2.79	0.466
amp FERG_wave_b µV	124.04 ± 42.41	134.94 ± 40.54	0.800
