# Supplementary material for: The Relative Preservation of the Central Retinal Layers in Leber Hereditary Optic Neuropathy
Source: J Clin Med. 2022 Oct 13;11(20):6045. doi: 10.3390/jcm11206045 (PMC9604528; doi:10.3390/jcm11206045)
Supplement: Supplementary file 1 [file jcm-11-06045-s001.zip › Supporting Table S5 JCM.pdf]

Table S5. The thickness of the different retinal layers for the selected eye for three ETDRS rings in LHON and nonLHON patients (mean and standard deviation-in brackets).

	LHON			NonLHON		
**variable**	**center**	**middle**	**outer**	**center**	**middle**	**outer**
GCC	36.9 (3.1)	54.9 (2.7)	51.3 (2.7)	29.1 (1.4)	81.6 (4.3)	63.3 (3.4)
INL	27.7 (10)	41.8 (7.3)	36.1 (3.7)	18.7 (4.6)	42.2 (6)	33.8 (2.7)
OPL	24.8 (4.9)	32 (5.6)	29.4 (2.6)	23.9 (2.5)	32.9 (3.4)	28 (2.9)
ONL	92.7 (10.1)	76.9 (8.4)	60.5 (7)	91.5 (13.1)	73.7 (9)	60.3 (6.6)
