# Supplementary material for: The Relative Preservation of the Central Retinal Layers in Leber Hereditary Optic Neuropathy
Source: J Clin Med. 2022 Oct 13;11(20):6045. doi: 10.3390/jcm11206045 (PMC9604528; doi:10.3390/jcm11206045)
Supplement: Supplementary file 1 [file jcm-11-06045-s001.zip › Supporting Table S6 JCM.pdf]

Table S6. Comparing mean ratios between different ETDRS regions for LHON versus nonLHON group using Welch's t-test

Ratio	Layer	LHON mean	Range	NonLHON mean	Range	t-value	*p* value
middle/center	retina	1.07	0.92-1.24	1.16	1.00-1.27	−2.68	**0.014**
	GCC	1.62	0.82-2.38	2.24	1.41-3.04	−3.18	**0.004**
	INL	1.83	0.84-3.16	2.40	1.14-3.30	−1.91	0.068
	OPL	1.35	0.91-1.85	1.39	1.10-1.80	−0.36	0.725
	ONL	0.83	0.69-0.98	0.81	0.61-0.89	0.63	0.536
outer/center	retina	0.99	0.85-1.07	1.05	0.97-1.13	−2.01	0.065
	GCC	1.85	0.85-2.58	2.41	1.91-3.15	−2.78	**0.013**
	INL	1.70	0.75-2.71	1.99	1.57-2.41	−1.16	**0.040**
	OPL	1.20	0.83-1.54	1.20	0.93-1.49	0.01	0.582
	ONL	0.66	0.51-0.80	0.65	0.50-0.72	0.30	0.974
outer/middle	retina	0.90	0.86-0.97	0.89	0.87-0.91	1.47	0.172
	GCC	1.05	0.92-1.25	1.01	0.81-1.18	0.24	0.810
	INL	0.83	0.68-0.88	0.78	0.72-0.82	2.28	**0.040**
	OPL	0.87	0.72-0.97	0.85	0.79-0.90	0.57	0.582
	ONL	0.80	0.74-0.85	0.80	0.73-0.85	0.03	0.974

Statistically significant p values are marked in bold
