# Supplementary material for: The Relative Preservation of the Central Retinal Layers in Leber Hereditary Optic Neuropathy
Source: J Clin Med. 2022 Oct 13;11(20):6045. doi: 10.3390/jcm11206045 (PMC9604528; doi:10.3390/jcm11206045)
Supplement: Supplementary file 1 [file jcm-11-06045-s001.zip › Supporting Table S7 JCM.pdf]

Table S7. Mean and standard deviation (in brackets) of retinal thickness for DOA patients (right and left eye)

**RE**	**Center**	**Middle**	**Outer**	**LE**	**Center**	**Middle**	**Outer**
retina	274.7 (6.51)	306.2 (11.86)	273.5 (8.88)	retina	276.3 (6.35)	308.9 (14.29)	275.8 (10.24)
GCC	11 (2.08)	22.23 (1.50)	22.03 (1.39)	GCC	10.9 (1.53)	22.93 (1.44)	23.16 (2.13)
INL	23.3 (4.04)	47.1 (3.64)	38.4 (4.19)	INL	22.0 (1.73)	47.7 (6.09)	37.8 (2.98)
OPL	24.7 (1.53)	33.8 (2.60)	29.2 (2.95)	OPL	27.7 (4.16)	32.2 (3.13)	28.5 (2.88)
ONL	102.3 (2.08)	75.9 (10.38)	60.4 (12.88)	ONL	104.3 (6.03)	78.2 (10.89)	61.8 (12.82)
